# Sonographic features differentiating early-stage ovarian clear cell carcinoma from endometrioma with atypical features

**DOI:** 10.1186/s13048-022-01019-8

**Published:** 2022-07-14

**Authors:** Kuan-Ju Huang, Ying-Xuan Li, Chin-Jui Wu, Wen-Chun Chang, Lin-Hung Wei, Bor-Ching Sheu

**Affiliations:** 1 National Taiwan University Hospital, Yunlin Branch, Yunlin county, Taiwan; 2 Graduate Institute of Clinical Medicine, National Taiwan University College of Medicine, Taipei, Taiwan; 3 Department of Obstetrics and Gynecology, National Taiwan University Hospital, National Taiwan University College of Medicine, 7 Chung-Shan South Road, Taipei, Taiwan; 4 National Taiwan University Hospital, Hsin-Chu Branch, Hsinchu City, Taiwan

**Keywords:** Endometriosis, Ovary, Clear cell carcinoma, Sonography

## Abstract

**Background:**

Ovarian clear cell carcinoma (OCCC) is the most common endometriosis-associated ovarian cancer. Ovarian endometriosis may present with atypical or malignant sonographic features and interfere with clinical judgment about whether definitive surgical intervention is required.

**Objective:**

To compare the characteristics of endometrioma with atypical features and OCCC.

**Methods:**

This study enrolled patients with pathologic diagnoses of either endometrioma or OCCC. For patients with endometrioma, only those with atypical features, defined as the presence of at least one of the following sonographic characteristics: cyst diameter of 10 ± 1 cm, multi-cystic lesions, any solid component or papillary structure, and blood flow of any degree, were included.

**Results:**

Sixty-three patients had endometriomas with atypical features, while 57 patients had OCCC. Patients with endometriomas were younger (39.33 ± 7.04 years vs. 53.11 ± 9.28 years, *P* < 0.01), had smaller cysts (7.81 ± 2.81 cm vs. 12.68 ± 4.60 cm, *P* < 0.01), and had smaller solid components (0.93 ± 1.74 cm vs. 4.82 ± 3.53 cm, *P* < 0.01). In contrast, OCCCs were associated with loss of ground-glass echogenicity (6.3% vs 68.4%, *P* < 0.01). In multivariate analysis, advanced age (> 47.5 years), large cysts (> 11.55 cm), large solid components (size > 1.37 cm), and loss of ground-glass echogenicity were independent factors suggestive of malignancy.

**Conclusion:**

Advanced age, larger cyst sizes, larger solid component sizes, and loss of ground-glass echogenicity are major factors differentiating endometriomas from malignancies. For women in menopausal transition who have finished childbearing who present with endometrioma with atypical features, removal of the adnexa intact could be considered.

**Supplementary Information:**

The online version contains supplementary material available at 10.1186/s13048-022-01019-8.

## Introduction

Endometriosis may affect up to 15% of women of reproductive age [1–3]. The adnexa and uterus are the most common organs involved. Besides its use during diagnosis, surgery is no longer the only management strategy for endometriosis due to concerns about fertility preservation and complications from repeat surgeries [4–7]. Many approaches have emerged to prevent recurrence [5, 6, 8, 9]. Surgery may be reserved for those with failed medical treatments or when a malignancy is suspected [5, 7]. Endometriosis-associated ovarian cancers (EAOC) present in 0.14%–2.9% of patients with endometriomas [10–14]. Among EAOCs, the most common histology is ovarian clear cell carcinoma (OCCC) [15].

EAOC occurs in women of older ages and is characterized by the presence of solid components and larger tumor sizes [10, 16, 17]. Sonography remains the first choice for assessment of the risk of malignancy. However, as age increases, endometriomas with retracted blood clots mimicking solid components become more common [7, 17, 18]. These lesions could also present with another atypical feature during the follow-up period, like large-sized or multi-cystic lesions [7, 18]. A search of the International Ovarian Tumor Analysis (IOTA) database revealed that up to 21% of endometriomas in women 45 years or older might have solid components [18]. Furthermore, since OCCC is usually diagnosed during its early stages, conventional image indicators for malignancy may have limited diagnostic value [9, 19]. Information about how to distinguish endometriomas from OCCCs is lacking and urgently needed [7]. This study aims to compare their characteristics with the goal of aiding clinical risk stratification.

## Materials and methods

This is a retrospective study of patients who were diagnosed with endometrioma or OCCC between January 2018 and December 2020 at National Taiwan University Hospital, one of the leading teaching hospitals in Taiwan, and a referral center for the diagnosis and treatment of gynecologic oncology. In our institution, all the ultrasound examiners were trained residents supervised by experienced examiners or attending physicians. The study was approved by the institute’s ethics committee (202111078RIN) and complies with the Declaration of Helsinki. The institution’s ethics committee waived the need for informed consent given the retrospective design of the study. Patients’ medical data were retracted from National Taiwan University Hospital-Integrated Medical Database specified with diagnoses of endometriosis and ovarian neoplasm. The diagnosis of an endometrioma could be based on clinical judgement or pathologic diagnosis. An endometrioma with atypical features is defined when at least one of the following sonographic features are present: cyst diameters of 10 ± 1 cm, multi-cystic lesions, any solid component or papillary structure, and blood flow of any degree. We followed the IOTA definition of a papillary projection, which is a protrusion of solid tissue into a cyst cavity with a height of at least 3 mm. A solid component was defined as a structure consisting of tissue. If it was unclear whether a structure consisted of solid tissue or amorphous material (such as a blood clot), the structure was classified as a solid component. If more than one adnexal mass was detected, the mass with the most complex ultrasound morphology was used for statistical analysis. If bilateral masses had similar ultrasound morphologies, the largest one, or the one most easily accessible by ultrasound, was used. The blood flow in a papillary structure observed by color or power Doppler was measured subjectively by the ultrasound examiner [20]. An IOTA risk score was assessed by simple rules and defined by five benign features—unilocular cysts, presence of solid components with maximum diameters < 7 mm, acoustic shadows, smooth multilocular tumors with maximum diameters < 10 cm, and no blood flow—and five malignant features—irregular solid tumors, ascites, at least four papillary structures, irregular multilocular, solid tumors with max diameter ≥ 10 cm, and very strong blood flow [21, 22]. Images with the most representative atypical or malignant features listed above were recorded without storage limitation. Generally, patients were referred to consultation for surgery and pathologic diagnosis when they had endometriomas with atypical features. In the study group, only patients receiving surgery within 180 days of ultrasound records of endometriomas with atypical features were included. Another group, the OCCC cohort, were enrolled in the same study period. All surgical specimens were evaluated by a pathologist specialized in gynecology. The baseline characteristics were recorded and analyzed. After diagnosis, the achieved images were retrospectively reviewed and analyzed. The authors were not blind to these images and their pathologic diagnosis.

## Statistical analysis

Statistical analyses were performed using SPSS Version 23.0 (SPSS Inc., Chicago, IL, USA). All data were compared by the Student’s *t* test, Fisher’s exact test, and the Chi‐square test. Multivariate analysis was performed by logistic regression analysis. To determine an optimal cut-off value for continuous variables, the receiver operating characteristic (ROC) curve was applied. A value of *P* < 0.05 was considered statistically significant for all tests.

## Results

**Fig. 1:**
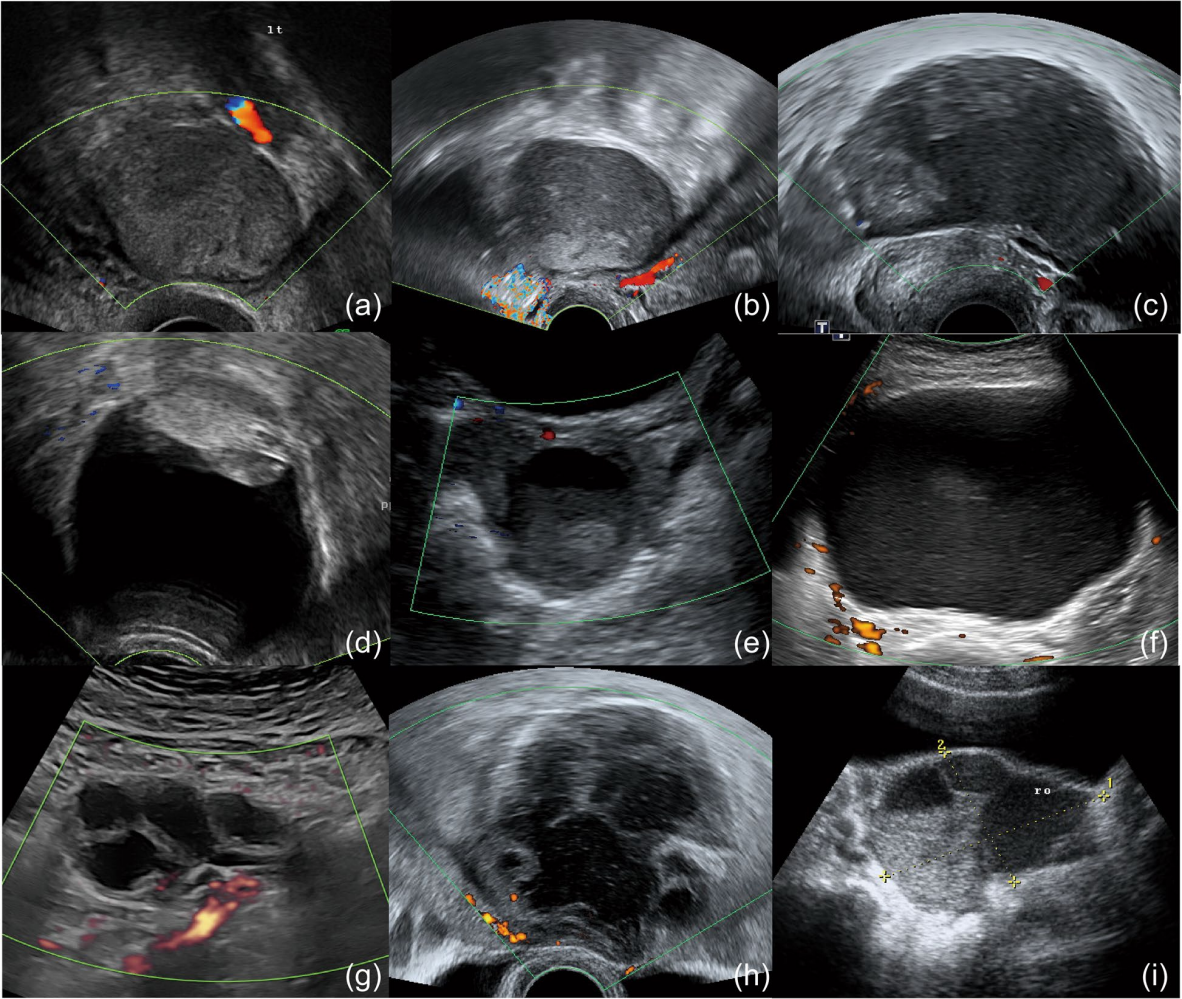
Endometrioma with atypical features. (**a**, **b**) Solid components and multilocular cysts, (**c**, **d**, **f**) floating tumors, (**d**, **e**, **f**) lesions wider than they are tall, and (**g**, **h**, **i**) multilocular cysts

**Fig. 2:**
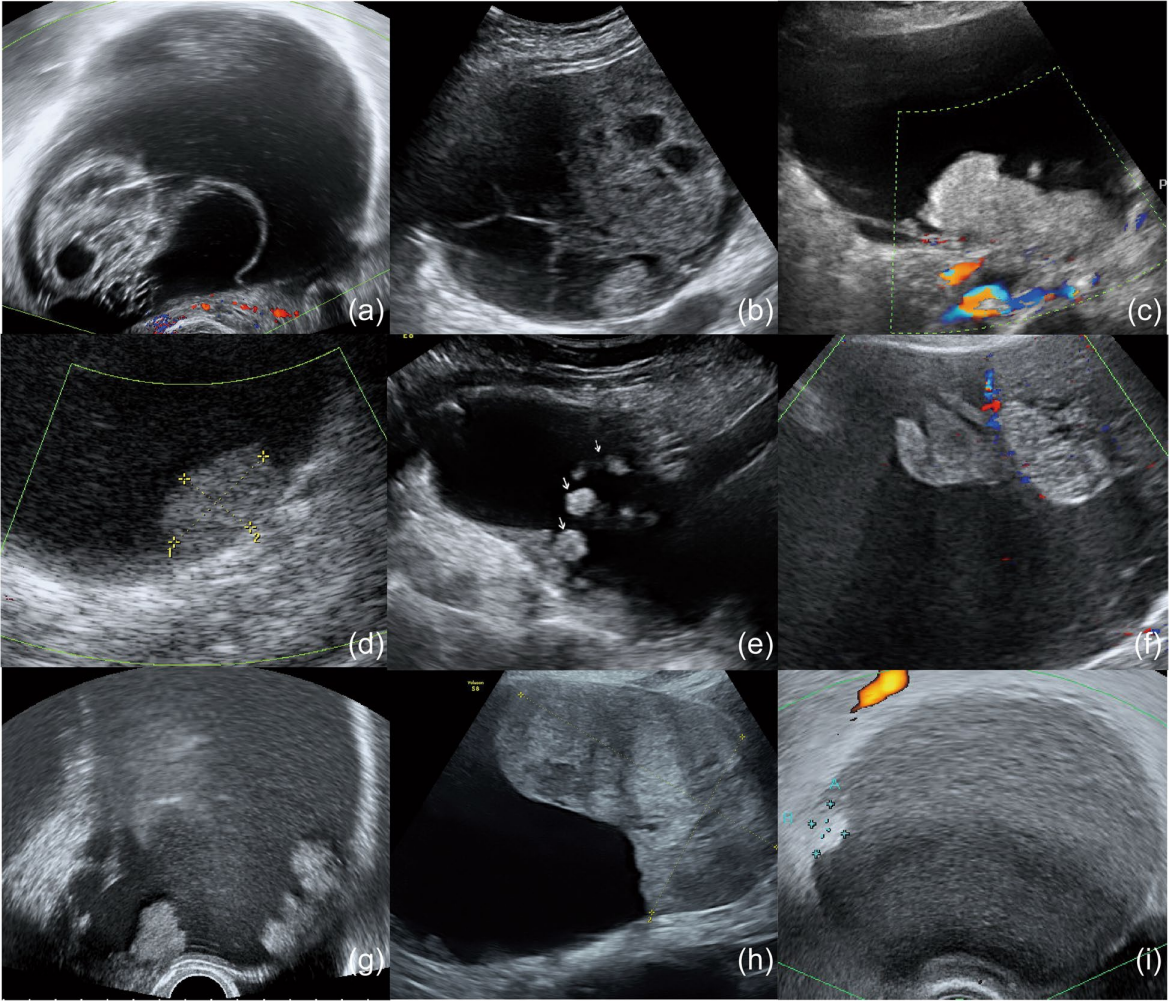
Ovarian clear cell carcinoma. (**a**, **b**) Solid components and multilocular cysts, (**c**, **d**) lesions wider than they are tall, (**e**, **f**) floating tumors, (**g**) solid components with papillary structure, and (**h**, **i**) solid components

There were 491 patients diagnosed with ovarian endometriosis, and 63 patients had atypical features in their endometriomas (12.83%) (Fig. 1). Fifty-seven patients had OCCCs (Fig. 2), which accounts for 16.76% of all types of epithelial ovarian cancers treated in our institution within the same study period. Patients’ baseline and sonographic characteristics are summarized in Table 1 and Table 2. In brief, patients with endometriomas were younger (39.33 ± 7.04 years vs. 53.11 ± 9.28 years, *P* < 0.01), had smaller cyst sizes (7.81 ± 2.81 cm vs. 12.68 ± 4.60 cm, *P* < 0.01), and had more bilateral involvement (28.6% vs. 1.8%, *P* < 0.01). The solid components were smaller (0.93 ± 1.74 cm vs. 4.82 ± 3.53 cm, *P* < 0.01) and fewer (< 4 lesions, 98.4% vs. 84.2%, *P* < 0.01) in endometriomas, and several of these lesions were wider than they were tall (12.7% vs. 0%, *P* < 0.01). In contrast, OCCCs were associated with loss of ground-glass echogenicity (6.3% vs. 68.4%, *P* < 0.01). The average IOTA risk for malignancy was 18.97 ± 29.75% and 44.74 ± 34.88% for endometriomas and OCCCs, respectively (*P* < 0.01). BMI, CA-125 indicating the presence of floating tumors, sediments, and other sonographic features were similar between groups.

**Table 1:** Baseline patient characteristics

(Mean, SD)	Endometrioma		OCCC		*P* Value	OR/Difference	95% CI	
	*N* = 63		*N* = 57				Lower	Upper
Age (years)	39.33	7.04	53.11	9.28	** < 0.01**	-13.77	-16.73	-10.81
BMI (kg/m^2^)	21.16	3.54	22.17	3.27	0.11	-1.01	-2.25	0.22
CA-125 (U/mL)	72.88	75.36	151.36	297.41	0.06	-78.48	-160.29	3.32
Endometriosis	63	100.00%	47	82.50%	** < 0.01**	1.21	1.08	1.37
Diameter (cm)	7.81	2.81	12.68	4.60	** < 0.01**	-4.88	-6.28	-3.48
Unilateral cysts	45	71.40%	56	98.20%	** < 0.01**	0.05	0.01	0.35
Loss of ground-glass echogenicity	4	6.3%	39	68.4%	** < 0.01**	0.03	0.01	0.10
Solid components								
Size (cm)	0.93	1.74	4.82	3.53	** < 0.01**	-3.90	-4.93	-2.87
Number (< 4 lesions)	62	98.4%	48	84.2%	** < 0.01**	11.63	1.42	94.94
Presence of sediments	9	14.30%	7	12.30%	0.75	1.19	0.41	3.44
Floating tumors	8	12.70%	5	8.80%	0.49	1.51	0.47	4.92
Lesions wider than they are tall	8	12.70%	0	0.00%	** < 0.01**	1.15	1.04	1.26

**Table 2:** IOTA rule and risk of ovarian cancer

(Mean, SD)	Endometrioma		OCCC		*P* Value	OR	95% CI	
	*N* = 63		*N* = 57				Lower	Upper
**Benign Features**								
Unilocular cysts (B1)	16	25.40%	45	78.90%	** < 0.01**	11.02	4.70	25.85
Solid components less than 7 mm (B2)	8	12.70%	2	3.5	0.10	0.25	0.05	1.23
Acoustic shadows (B3)	0	0.00%	0	0.00%	-	-	-	-
Smooth multilocular tumors less than 10 cm (B4)	29	46.00%	0	0.00%	** < 0.01**	0.54	0.43	0.68
No Blood flow (B5)	54	85.70%	30	52.60%	** < 0.01**	0.19	0.08	0.45
**Malignant Features**								
Irregular tumors (M1)	24	38.10%	56	98.20%	** < 0.01**	91.00	11.81	701.05
Ascites (M2)	1	1.60%	3	5.30%	0.35	3.44	0.35	34.10
Tumors with more than 3 papillary projections (M3)	1	1.60%	9	15.80%	** < 0.01**	11.63	1.42	94.94
Multilocular tumors > 10 cm with solid components (M4)	2	3.20%	8	14.00%	**0.05**	4.98	1.01	24.53
Strong blood flow (M5)	9	14.30%	27	47.40%	** < 0.01**	5.4	2.25	12.97
**IOTA Simple Rule Risk**	18.97%	29.75%	44.74%	34.88%	** < 0.01**	-0.27	-0.39	-0.15

Table 3 shows the multivariate analysis results for malignancy. Age (*P* < 0.01; OR, 1.34; 95% CI, 1.11–1.62), cyst diameter (*P* < 0.01; OR, 1.58; 95% CI, 1.12–2.24), loss of ground-glass echogenicity (*P* = 0.02; OR, 27.03; 95% CI, 1.69–433.75), and solid-components size (*P* < 0.01; OR, 2.15; 95% CI, 1.23–3.75) were independent factors suggestive of malignancy. However, the IOTA risk score had a limited role in distinguishing endometriomas from OCCCs (*P* = 0.89; OR, 1.26; 95% CI, 0.05–30.07). In the subgroup analysis, 19.37% (11/57) patients < 45 years of age had OCCCs. Cyst diameter (*P* < 0.01; OR, 1.58; 95% CI, 1.12– 2.24) and loss of ground-glass echogenicity (OR, 27.03; 95% CI, 1.69–433.75) remain factors suggestive of malignancy (Supplementary Tables 1, 2, and 3). Another subgroup, defined by only four variables, multi-cystic lesions, any solid component or papillary structure, and blood flow of any degree, resulted in similar findings that age (*P* < 0.01; OR, 1.22; 95% CI, 1.08–1.37), cyst diameter (*P* = 0.01; OR, 1.37; 95% CI, 1.07–1.75), solid-component size (*P* < 0.01; OR, 1.77; 95% CI, 1.17–2.68), and loss of ground-glass echogenicity (*P* = 0.01; OR, 13.28; 95% CI, 1.74–101.61) were factors suggestive of malignancy (Supplementary Tables 4, 5, and 6).

**Table 3:** Multivariable analysis

(Mean, SD)	Univariate	Multivariate			95% CI	
	*P* Value	B	*P* Value	OR	Lower	Upper
Age	** < 0.01**	0.29	** < 0.01**	1.34	1.11	1.62
CA-125	**0.05**	< 0.01	0.52	1	0.99	1.02
Endometriosis	1.00	-	-	-	-	-
Diameter	** < 0.01**	0.46	** < 0.01**	1.58	1.12	2.24
Unilateral cysts	** < 0.01**	-2.45	0.16	0.09	< 0.01	2.53
Loss of ground-glass echogenicity	** < 0.01**	3.3	**0.02**	27.03	1.69	433.75
Solid components						
Size (cm)	** < 0.01**	0.77	** < 0.01**	2.15	1.23	3.75
Number (< 4 lesions)	** < 0.01**	3.7	0.06	40.55	0.86	1908.57
Lesions wider than they are tall	1.00	-	-	-	-	-
IOTA Simple Rule Risk	** < 0.01**	0.23	0.89	1.26	0.05	30.07
Constant	-	-21.3	< 0.01	0	-	-

The ROC curve and the corresponding sensitivity, specificity, and positive and negative predictive values for age, cyst size, and solid-component size are listed in Table 4. In general, age > 47.5 years, cyst size > 11.55 cm, and solid-component size > 1.37 cm may indicate the optimal cut-offs for surgical management of patients presenting with endometriomas with atypical features (Figure S1).

**Table 4:** Diagnostic values for age, cyst diameter and size of solid components

Variables	Cut-off	Sensitivity	Specificity	PPV	NPV	AUC
Age	47.5 years	77.2%	93.7%	92%	80%	0.875
Cyst Diameter	11.55 cm	63.2%	95.2%	93%	72%	0.814
Size of Solid Component lesions	1.37 cm	89.5%	81%	82%	89%	0.892

## Discussion

Endometriosis is considered a chronic disease [4–6]. Previously, surgical intervention was the choice for diagnosis and primary treatment of endometriomas. The postoperative recurrence rate for endometrioma ranged from 2%–30% within 3 years [8]. With increased concerns about fertility outcomes, conservative treatment is becoming the preferred option unless treatment fails or malignancy is suspected [5, 7]. OCCCs could account for 76% for EAOCs, and they make up 20% of epithelial ovarian cancers in Asian countries, with the highest rates reported in Taiwan, Japan, and Thailand [15, 23]. For risk assessments of ovarian malignancy in clinical practice, the IOTA rule is an effective risk estimation method [22]. However, with increasing age, endometriomas are more likely to present as multilocular cysts, cysts with solid components, and cysts with decreased ground-glass echogenicity [18]. In addition, most OCCCs lack ascites at the time of diagnosis, and the pre-operative CA-125 serum values was not significantly different between endometriosis patients with or without EAOCs [10, 16, 19]. These factors influence diagnostic value of the IOTA method and confuse examiners who are making conclusions. More parameters might be helpful when providing consultations for these patients with high-risk sonographic features.

In the current study, we found an increased risk of OCCC in women of advanced age. Our analysis showed a cut-off of 47.5 years old in endometrioma patients with atypical features, while previous findings suggested women 43 to 45 years or older were at higher risk [10, 16, 17]. A large cyst, which ranges from 4.2 cm to 9 cm in other reports and was 11.5 cm in the current study, is also an independent factor for suspicion of malignancy [10, 16]. An age-stratified strategy has been used for screening patients at higher EAOC risk [7, 10, 15–17]. Some studies also revealed a reduced risk of EAOC by surgical intervention for patients of advanced age (≥ 45 years) and endometriomas ≥ 8 cm [7, 9, 16].

Papillary projections and solid components are some of the most identifiable characteristics for differentiating malignancies from benign lesions and for making the decision to intervene surgically [22]. However, retracted blood clots sometimes simulate solid components that are indistinguishable from malignancies. Although most blood clots can precipitate to dependent sites, float within chocolate-like cyst fluid, or be less confined to the cyst wall, limited evidence is available to describe benign solid components [17]. In one study evaluating adnexal lesions by magnetic resonance imaging, heights > 1.5 cm or height-to-width ratios of mural nodules > 0.9 were suggestive of malignancies [17]. We had similar results using sonography; however, multivariate analysis indicated that this factor is insufficient for decision-making (*P* = 1.00). The presentation of this feature by a small percentage of benign lesions (12.7%) and the effect of subjective recordings of sonographic images could explain this inconsistency. However, we found that the larger the size of the solid component, the higher risk of malignancy, especially when > 1.37 cm. There may be no other evidence to support this finding, but blood clots may degrade with time, while tumors would grow indefinitely.

Another major factor identified to distinguish endometriomas from OCCCs is the loss of ground-glass echogenicity. It could be a normal presentation in women ≥ 45 years [18]. It had been hypothesized that the cysts in an endometrioma progressively dissolve because of the effects of local degrading enzymes present at high levels in these lesions [18, 24]. Cyclic bleeding may replace the degraded cyst content, again presenting as classic ground-glass echogenicity. When in menopausal transition, there is less fresh blood for the generation of sonography signals. However, after adjusting for age, or in patients < 45 years of age, the loss of ground-glass echogenicity still plays an important role in the identification of malignancies. More studies are required to determine whether loss of ground-glass echogenicity represents an aged endometrioma or increased activity of local degrading enzymes in OCCC.

This study is limited by its retrospective design. There could be inherent bias due to the regular follow-ups for patients with endometriomas compared with fewer follow-ups for patients with OCCCs. Second, despite standard training and evaluation by senior examiners, there could be inter-observer variation or errors in the recording of sonography images. Third, although endometrioma and OCCC are closely correlated, our results were not sufficient to provide strong evidence for use in the clinical decision-making process, especially for patients < 45 years of age, in whom ovarian preservation is still a concern. Fourth, being an referral cancer center, the prevalence of OCCC might be overestimated, especially the study period overlapped with the pandemic when elective surgery for benign disease might be deferred.

In conclusion, our findings are in line with previous studies in that advanced age and larger cyst size are major factors differentiating endometriomas from malignancies. We also discovered that larger solid-component size and loss of ground-glass echogenicity could play important roles and aid in decision-making. For women in menopausal transition and those who have completed childbearing presenting with endometriomas with atypical features, removal of adnexa intact could be considered. However, more research is required to provide guidance to younger women, including consideration of fertility-sparing management strategies.

## Supplementary Information

**Additional file 1:** **Supplementary Table 1.** Baseline Characteristics forPatients ≤45 years of Age. **SupplementaryTable 2.**IOTA Rule and Risk for Ovarian Cancer for Patients ≤45years of Age. **SupplementaryTable 3.**Multivariable Analysis for Patients ≤45 years of Age. **Supplementary Table 4**. Baseline Characteristics for AtypicalFeatures Excluding Cyst Diameter Criteria. **SupplementaryTable 5**.IOTA Rule and Risk for Ovarian Cancer for AtypicalFeatures Excluding Cyst Diameter Criteria. **SupplementaryTable 6**Multivariable Analysis for Atypical Features Excluding Cyst Diameter Criteria.

**Additional file 2:** **Figure S1. **ROCcurve for age, cyst diameter, and size ofsolid components.
