# Supplementary material for: Sonographic features differentiating early-stage ovarian clear cell carcinoma from endometrioma with atypical features
Source: J Ovarian Res. 2022 Jul 14;15:84. doi: 10.1186/s13048-022-01019-8 (PMC9284754; doi:10.1186/s13048-022-01019-8)
Supplement: Supplementary file 1 — Additional file 1: Supplementary Table 1. Baseline Characteristics forPatients ≤45 years of Age. SupplementaryTable 2.IOTA Rule and Risk for Ovarian Cancer for Patients ≤45years of Age. SupplementaryTable 3.Multivariable Analysis for Patients ≤45 years of Age. Supplementary Table 4. Baseline Characteristics for AtypicalFeatures Excluding Cyst Diameter Criteria. SupplementaryTable 5.IOTA Rule and Risk for Ovarian Cancer for AtypicalFeatures Excluding Cyst Diameter Criteria. SupplementaryTable 6Multivariable Analysis for Atypical Features Excluding Cyst Diameter Criteria. [file 13048_2022_1019_MOESM1_ESM.docx]

**Supplementary Table 1 Baseline Characteristics for Patients ≤45 years of Age**

| (Mean, SD) | Endometrioma | | OCCC | | P Value | Difference/OR | 95% CI | |
| --- | --- | --- | --- | --- | --- | --- | --- | --- |
|  | 49 | | 11 | |  |  | Lower | Upper |
| Age | 36.9 | 5.68 | 38.18 | 5.81 | 0.50 | 1.28 | -2.53 | 5.09 |
| BMI | 21.48 | 3.89 | 22.45 | 3.4 | 0.76 | 0.96 | -1.58 | 3.51 |
| CA-125 | 68.22 | 71.51 | 105.6 | 117.72 | 0.19 | 37.38 | -19.33 | 94.08 |
| Endometriosis | 49 | 100% | 10 | 90.90% | 0.18 | 0.91 | 0.75 | 1.10 |
| Diameter | 7.97 | 2.89 | 12.87 | 4.93 | **<0.01** | 4.90 | 1.53 | 8.27 |
| Unilateral cysts | 33 | 67.30% | 11 | 100% | **0.03** | 1.49 | 1.22 | 1.80 |
| Loss of ground-glass echogenicity | 3 | 6.10% | 7 | 63.60% | **<0.01** | 26.83 | 4.93 | 146.16 |
| Solid components |  |  |  |  |  |  |  |  |
| Size (cm) | 0.99 | 1.88 | 3.68 | 2.46 | **<0.01** | 2.69 | 1.36 | 4.03 |
| Number (<4 lesions) | 48 | 98% | 9 | 81.80% | 0.08 | 0.09 | <0.01 | 1.15 |
| Presence of sediments | 5 | 10.20% | 0 | 0.00% | 0.57 | 1.11 | 1.01 | 1.22 |
| Floating tumors | 8 | 16.30% | 1 | 9.10% | 1.00 | 1.95 | 0.22 | 17.45 |
| Lesions wider than they are tall | 7 | 14.30% | 0 | 0.00% | 0.33 | 1.17 | 1.04 | 1.31 |

**Supplementary Table 2 IOTA Rule and Risk for Ovarian Cancer for Patients ≤45 years of Age**

| (Mean, SD) | Endometrioma | | OCCC | | P Value | Difference/OR | 95% CI | |
| --- | --- | --- | --- | --- | --- | --- | --- | --- |
|  | 49 | | 11 | |  |  | Lower | Upper |
| **Benign Features** |  |  |  |  |  |  |  |  |
| Unilocular cysts (B1) | 13 | 26.50% | 9 | 81.80% | **<0.01** | 0.08 | 0.02 | 0.42 |
| Solid components less than 7mm (B2) | 4 | 8.20% | 1 | 9.10% | 1.00 | 0.89 | 0.09 | 8.83 |
| Acoustic shadows (B3) | 49 | 100.00% | 11 | 100.00% | - | - | - | - |
| Smooth multilocular tumors less than 10cm (B4) | 24 | 49.00% | 0 | 0.00% | **<0.01** | 1.96 | 1.49 | 2.58 |
| No Blood flow (B5) | 43 | 87.80% | 5 | 45.50% | **<0.01** | 8.6 | 1.99 | 37.11 |
| **Malignant Features** |  |  |  |  |  |  |  |  |
| Irregular tumors (M1) | 17 | 34.70% | 11 | 100.00% | **<0.01** | 2.88 | 1.96 | 4.23 |
| Ascites (M2) | 1 | 2.00% | 0 | 0.00% | 1.00 | 1.02 | 0.98 | 1.06 |
| Tumors with more than 3 papillary projections (M3) | 1 | 2.00% | 2 | 18.20% | 0.08 | 0.09 | <0.01 | 1.15 |
| Multilocular tumors >10 cm with solid components (M4) | 2 | 4.10% | 1 | 9.10% | 0.46 | 0.43 | 0.04 | 5.16 |
| Strong blood flow (M5) | 6 | 12.20% | 6 | 54.50% | **<0.01** | 0.12 | 0.03 | 0.5 |
| **IOTA Simple Rule Risk** | 16.00% | 27.60% | 47.00% | 38.10% | **<0.01** | -31.00% | -11.00% | -51.00% |

**Supplementary Table 3 Multivariable Analysis for Patients ≤45 years of Age**

| (Mean, SD) | P Value | Multivariate | | | | 95% CI | |
| --- | --- | --- | --- | --- | --- | --- | --- |
|  | Univariate | B | P Value | OR | | Lower | Upper |
| Age | 0.50 | - | - | | - | - | - |
| BMI | 0.45 | - | - | | - | - | - |
| CA-125 | 0.21 | - | - | | - | - | - |
| Endometriosis | 1.00 | - | - | | - | - | - |
| Diameter | **<0.01** | -0.3 | **0.05** | | 0.74 | 0.55 | 0.99 |
| Unilateral cysts | 1.00 | - | - | | - | - | - |
| Loss of ground-glass echogenicity | **<0.01** | 3.13 | **0.02** | | 22.89 | 1.79 | 292.16 |
| Solid components |  |  |  | |  |  |  |
| Size | **<0.01** | -0.14 | 0.59 | | 0.87 | 0.53 | 1.43 |
| Number | 0.06 | - | - | | - | - | - |
| Presence of sediments | 1.00 | - | - | | - | - | - |
| Floating tumors | 0.55 | - | - | | - | - | - |
| Lesions wider than they are tall | 1.00 | - | - | | - | - | - |
| IOTA Simple Rule Risk | **<0.01** | -3.24 | 0.06 | | 0.04 | <0.01 | 1.11 |
| Constant | - | 3.63 | 0.06 | | 37.69 | - | - |

**Supplementary Table 4 Baseline Characteristics for Atypical Features Excluding Cyst Diameter Criteria**

| (Mean, SD) | Endometrioma | | OCCC | | P Value | Difference/OR | 95% CI | |
| --- | --- | --- | --- | --- | --- | --- | --- | --- |
|  | 58 | | 57 | |  |  | Lower | Upper |
| Age | 39.45 | 7.03 | 53.11 | 9.28 | **<0.01** | -13.66 | -16.69 | -10.62 |
| BMI | 21.05 | 3.56 | 22.17 | 3.27 | 0.08 | -1.12 | -2.38 | 0.14 |
| CA-125 | 71.34 | 72.62 | 151.36 | 297.41 | 0.06 | -80.03 | -161.82 | 1.77 |
| Endometriosis | 58 | 100% | 47 | 82.5% | **<0.01** | 0.83 | 0.73 | 0.93 |
| Diameter | 7.57 | 2.79 | 12.68 | 4.60 | **<0.01** | -5.12 | -6.53 | -3.71 |
| Unilateral cysts | 41 | 70.7% | 56 | 98.2% | **<0.01** | 0.04 | <0.01 | 0.34 |
| Loss of ground-glass echogenicity | 4 | 6.9% | 39 | 68.4% | **<0.01** | 0.03 | 0.01 | 0.11 |
| Solid components |  |  |  |  |  |  |  |  |
| Size | 1.00 | 1.80 | 4.82 | 3.53 | **<0.01** | -3.82 | -4.86 | -2.78 |
| Number | 1 | 1.7% | 9 | 15.8% | **<0.01** | 0.09 | 0.01 | 0.77 |
| Presence of sediments | 9 | 15.5% | 7 | 12.3% | 0.62 | 1.31 | 0.45 | 3.80 |
| Floating tumors | 8 | 13.8% | 5 | 8.8% | 0.40 | 1.66 | 0.51 | 5.43 |
| Lesions wider than they are tall | 8 | 13.8% | 0 | 0% | **<0.01** | 1.16 | 1.05 | 1.29 |

**Supplementary Table 5 IOTA Rule and Risk for Ovarian Cancer for Atypical Features Excluding Cyst Diameter Criteria**

| (Mean, SD) | Endometrioma | | OCCC | | P Value | Difference/OR | 95% CI | |
| --- | --- | --- | --- | --- | --- | --- | --- | --- |
|  | 58 | | 57 | |  |  | Lower | Upper |
| **Benign Features** |  |  |  |  |  |  |  |  |
| Unilocular cysts (B1) | 11 | 19.0% | 45 | 78.9% | **<0.01** | 0.06 | 0.03 | 0.16 |
| Solid components less than 7mm (B2) | 8 | 13.8% | 2 | 3.5% | 0.09 | 4.41 | 0.89 | 21.74 |
| Acoustic shadows (B3) | 0 | 0.0% | 0 | 0.0% | - | - | - | - |
| Smooth multilocular tumors less than 10cm (B4) | 29 | 50.0% | 0 | 0.0% | **<0.01** | 2.00 | 1.55 | 2.58 |
| No Blood flow (B5) | 49 | 84.5% | 30 | 52.6% | **<0.01** | 4.90 | 2.03 | 11.76 |
| **Malignant Features** |  |  |  |  |  |  |  |  |
| Irregular tumors (M1) | 24 | 41.4% | 56 | 98.2% | **<0.01** | 0.01 | <0.01 | 0.10 |
| Ascites (M2) | 1 | 1.7% | 3 | 5.3% | 0.36 | 0.32 | 0.03 | 3.13 |
| Tumors with more than 3 papillary projections (M3) | 1 | 1.7% | 9 | 15.8% | **<0.01** | 0.09 | 0.01 | 0.77 |
| Multilocular tumors >10 cm with solid components (M4) | 2 | 3.4% | 8 | 14.0% | 0.05 | 0.22 | 0.04 | 1.08 |
| Strong blood flow (M5) | 9 | 15.5% | 27 | 47.4% | **<0.01** | 0.20 | 0.08 | 0.49 |
| **IOTA Simple Rule Risk** | 20.00% | 30.50% | 45.00% | 34.90% | **<0.01** | -25.20% | -37.30% | -13.10% |

**Supplementary Table 6 Multivariable Analysis for Atypical Features Excluding Cyst Diameter Criteria**

| (Mean, SD) | P Value | Multivariate | | | | 95% CI | |
| --- | --- | --- | --- | --- | --- | --- | --- |
|  | Univariate | B | P Value | OR | | Lower | Upper |
| Age | **<0.01** | 0.20 | **<0.01** | | 1.22 | 1.08 | 1.37 |
| BMI | 0.09 | - | - | | - | - | - |
| CA-125 | 0.10 | - | - | | - | - | - |
| Endometriosis | 1.00 | - | - | | - | - | - |
| Diameter | **<0.01** | 0.31 | **0.01** | | 1.37 | 1.07 | 1.75 |
| Unilateral cysts | **<0.01** | -2.47 | 0.09 | | 0.09 | <0.01 | 1.47 |
| Loss of ground-glass echogenicity | **<0.01** | 2.59 | **0.01** | | 13.28 | 1.74 | 101.61 |
| Solid components |  |  |  | |  |  |  |
| Size | **<0.01** | 0.57 | **<0.01** | | 1.77 | 1.17 | 2.68 |
| Number | **0.03** | 2.89 | 0.07 | | 18.07 | 0.82 | 395.84 |
| Presence of sediments | 0.62 | - | - | | - | - | - |
| Floating tumors | 0.40 | - | - | | - | - | - |
| Lesions wider than they are tall | 1.00 | - | - | | - | - | - |
| IOTA Simple Rule Risk | **<0.01** | -0.04 | 0.98 | | 0.96 | 0.08 | 12.33 |
| Constant | - | -14.07 | <0.01 | | <0.01 | - | - |
