# Supplementary material for: Sonographic features differentiating early-stage ovarian clear cell carcinoma from endometrioma with atypical features
Source: J Ovarian Res. 2022 Jul 14;15:84. doi: 10.1186/s13048-022-01019-8 (PMC9284754; doi:10.1186/s13048-022-01019-8)
Supplement: Supplementary file 2 — Additional file 2: Figure S1. ROCcurve for age, cyst diameter, and size ofsolid components. [file 13048_2022_1019_MOESM2_ESM.doc]

ôQïÞ½ï»ï¾Ó§Os UZÔª°fÍ%!ñÊ9ö¬§§§þMT&5w½¼¼ôËøûûÿóÏ?úó5¦üm·Ý&9ÖÖÖbÁ-[ª:ZU¦Ôðý÷ß«|	|56vïÞ]å÷ë×O½/$Zeï UÓ®];Í+ª$*UÀNåÆfÏ­ºP±¬¯¯ïöíÛµ§:þüã?®]¡Y³fÓ¦Mûë¯¿ªuµå;¦t(þRHª­ÕO>ùD§ÚnÝº©'¾Ú¢ØaÃ©Ì2ÇñãÇ9s½Vê¼¼¼ôôtõnhUdeeýöÛoÆ÷Ah¸|vvv~~¾I¾¸¸øÈ#êÅ!@«h­Ö¾(ÉÇ&­ÅO?ýæîî¾uëVý±Gñññqtt=ztaa!ûÐª!ÒÒÒ>ÿüsûJµ:dÈ¥KÌ?ýhµzÚµk§¯Õ¢¢"Ñ­üJzË-X½zõ¸3vìØï½÷y3úôÀBCCì±ÀËÔ`ð[nñóó»é¦>qâDí±&Lðôô¼á:uê$cÑZ5_­&''»¹¹©tVVVóæÍ/ ¯Õ1cÆX[[shlDFFÎªàá_íQ28jÔ(++«¾ûjR2»víÚ½÷6mÚxxxh´wïÞ¶¶¶×^í5×êêª­Õ=ÚVÐ¾ûÁkkUNA.´lÙRÆ¢´j¾Z]¿~.]T:;;»I&çÎÓöþÿ»À¬ÈÉÉÑy§6///..Nõ9¬:ú× aÑù´BBûeÂÃÃµGmÜ¸QåûûûkºcTìÚµKª9r¤OOÁÔ2lÚ´)33SçYfÆ¿X¬Íý÷ßïååÅ®G«æ«Õ³gÏÚÛÛ«÷Óå_çëë[é´h ÆªÁDx¢"íQéééó*>ºÕ%BR±D(âããUOíé¹Hl§Ü©S¡LÓË¿ÎbhT'¨GBÆ#èø»VA«hÕLµZVV¶lÙ²ÃKZT(3gÎ3­BcÀÕM<YÇLR¾*ÕiúÓ¯Tu:jæ£³%ÌÈÈ0U7OµHZV¡qiõí·ßîØ±£xÑÉÉIuUZRR¢ù>srr²³³³¬ÇG«P_hb¦ÔÔT;2¥¤s¿Q#-Aç.¥&ªÓéÍ_;ÕéÜÕ¨.66¶ª­Z­g=j G´Úh+°ªT§y~&ÂÓù"·zê&EGGkÒ<«³±±5kö(ñ¥æ1ÎTR¡Êv:óÊËËK¹ÎZ´j¦ UÀ@T§­:¨®Ú+Ï@	ì*õtÍZ¬X0²%ë:ØÙÙ988xxx8ð`×®]ôèQgiee%sähA«hêÜÜO;ªKOOGufÅ´iÓäÂ%¥^¹á|ðA9lÖ­[÷ÁD_¦ÚÁº¹ì´¼ ¬¬lË-?üðÃ6C«hjDº¸¸DDD°5óæÍ0`@AAAý.ÿuÕë¯¿þÿüç&ÉÉÉyé¥:qâÄeËáJ:uÊÕÕµcÇò·³ò@«`vçh!äÉ¦h,Z´¨W¯^æ°¿BCC÷ìÙcaZ(ÐÃÃÃÓÓSþwßwÿþýÍp¥V¯^mgg'Êß­ÀU'aSæ°0)))¦Õ¯¾úÊÊÊêÇTuð©®Ìâ±ÇK+þhêôôôé°)"â0wxÐªiµºmÛ6Ñê/¾¨/2÷ÜsOÆsâÄ	ïïï£ê&]¸óÎ;çÎß÷µlÙòØ±cÇ?~|»ví7oÞ½÷âââòþ_nkkëîî ¿0Îô^°··oÖ¬Ô£Ó*Ð*ÔEEE#Grdd¤Ä:pEìÙ³GN¾:/Õ/AAA§U!88XÌ*ÿM£ôÒÒÒ~ýúõèÑcÝºuâ?Iôïß_ÝíÐ¡Ã¼yóT1ùsuîÜY¥%´¶¶8q¢¿çÎóöönÛ¶í«¯¾zèÐ¡ÄÄD©P&ïÛ·¯lÃ~úiÆòßÔYªf=zôh	©«ê9Ð*ÔQ¬C£Á:»Ù´idådên"äDjV«i©MD]QQQ-*øà$gÇ²ýW¬X¡,_¾ÕmZ=«tZZ×é<Ye.Z´HâãO?ýTÒ÷îÕ.``¦Ó¦MëÓ§4´uJ~lºdÿþýK,øÃÁÁ!00PÎ¶ªãxknëkM4üöÛoó÷ßÿøãEi?ÿü³%nÁøøxÃZÕ<mQKØª]¿ÊìÒ¥KÏË|öÙgú*)ZE«P§¨¨...>­Ó"¶KJJ<y²²åE¥ä®E>[Õfûöíb²ØØØ­[·Jâý÷ßWù«V­A«´úÌ3Ï¨ü	&TªUUÏÜ¹sõ£U©¼ª¹)ZE«PwÈiÎÕÕ5&&¨µ£øùùÉÖÞûA´H­&''¯X±BöfaaáO<Ñ¼yó£GKXéïïðàÁ½÷Ê~÷ððPÍn¹åJæO>)Ú«T«%%%bA¹ÒG:uêË/¿<þ¼dzwïÞýo¾ª¤	µÄÀLÑ*Z°222D#G´³³fDDDjjjcîgÊ",IP(*A6mÚT´÷Þï©|[Ï=Õgúöí»oß>Ø¾kkkÙ3fÌÐhµwïÞ2¨ðøúúªÉ;vì(r-¯ø D@@äÈämÚ´Q_Ó¦ª>úè£2È_­éÉÍÍ"0­=òòòâããÃÃÃ===%ÚÁzïÞÈ|6E6Y*--=|øpvv¶þ¨?þøãÏ?ÿÔÉ¼xñâ_ýeäÓ/yöìÙ'OªÒZ ý±höGû÷ï<y²èÍ°1KCG6¯J`*QÈ¼yóÒÒÒ,;03u¤©«4ceËXp%@«hÕÕ¼_¡yå1SDDqjmª¾·ÐSõQX	ÇÕ&	É4fP¶RcÐjFFáhÐ*Z5÷cêõE<Ö×2¸z­j7>ºRÎ;÷â/Þï½²ÃÂÂ¢¢¢;Æ_­¢Õ:Eþx×àÍ°¾u¯ÕVÓÓÓ%¦···1cÆ+¯¼2eÊ:|úé§üÅÐ*Z­SæUÀv@3¬oiõæoÖþD«a­è(ÿ¶ÛnkÝºõÁ59eee¦z§¹¾VÑ*Z4S222,¸Çö+Õê9s7oÞªU«îÝ»ÇÅÅ)­>úè£³gÏvvvîÛ·ïæÍUIÃåÿúë¯VVV"t0¦~ÉÔL"j°Úú­Â¿?[tt4Û­ÖªÉZU;v¬wïÞ#FøñÇO>­´jcc#¡çç~ã78PÅ;ÊOJJ­~ûí·ÎÅúÅ¯mÚ´Qý?¤%ëÖ­ÕÎÐjcGø¸ù 'D	&t2e¤ÿþý3(çwooo´ªAÿ&ðàÁ/^¼(é_|±EåFtÿî»ïJ¦ÈïJµªé ?33STºvíZI÷ìÙÓùZmì5Jþ¢¸FBNNÓ´iÓt2ÝÝÝÓÓÓ/a1âTõE«U=[VZ­¶£üï¾ûNèd£Uíç¸CÀTVGGÇ7ß|ÓùZmìÈ¿¡qqq...Ë/×É¸A´Êöi<ZàkµÚòóòò¤¤x±*­VÛA¿ðáJÀºpáÂk¯½öÌ3ÆÌÐ*YPTT4vìXq§¶ôÇ¢ÕF¥ÕÐÐÐnÝºi^¯T«Õv/<ÿüó¢À'Êñ£>Áþý÷ß×A¿:,¤ÀÃ?¬r/ UzfÏ=rª5jTU*Iä¡yâ¯ÕíÛ·»¹¹ÙØØ¬²üßæKm8j.¶w_VV&nÝºµê+_ríÞ½»ÜèúË+zÕiüñGí<Ãó´PÄÆÆ:99­^½Úp1Ú5­W¼]ZZZmÉj;ÊªDËjçßAÍæVÑ*@]#±iHHÈÒÓÓTMØbG«VÑjcùòåó.ÃWÆêÔÔT1eXX~¯7¹¹¹½³qãÆýû÷K1+++6Z´VÑ*TBdd¤êCG	^]\´µ*	ýæÁV­¢Uhìäåå5Êßß?##£ª2r¡#*e[¡U@«hÕÜIIIá£oõ»ý]]]%ô4üAòÆÖy!Z´V*ô¦T_GÃÃÃ=<<Ùþh­ZE«hª$###((ÈÛÛÛÈdÐ*Z´VÑ*TNRRÃôéÓoÁ¦C«VÑj"ê²ÿªFEGGgffrÄ×³fÍrqqÙ¸q£áØÔÝÝ]~eïhº¡çê­Ö´´´Á8p-VÑêª>°%	ãGA±hÑ"??¿j¯cV¯^íéé§ùHßéC«5fâÄ;w~ì±ÇØòh­E!²ôððÈÍÍ5¦dXX[­^=gÏmÙ²åZ·n­yè°fÍ;î¸#::zÞ¼yêÛ¨?ÿüóäÉôQZ´V¡aäîînäýöýû÷Ky6Z½zÞyçÁK¢ÿþ+V¬ÄW_Õ±cÇÈÈH++«¯¿þ:++ËÁÁáÓO?÷ÝwÎ;Ç>B«hÌM6¹¸¸ìÙ³ÇÈòééé¾Åk5>>Þª¦ôêÕKSßc=öÝwß=ôÐCÞÞÞ3eÊ'|²¼¢ÃgggÑê%K|||VÐ£G?üVÑêÿ¾#Êk»ÆÕÕµÒo¦V7ÑªI8xð ­­íÝiÖ¬â×gVèÞ½»hõñÇ¿é¦^¼8VÑ*ï5)wS¯4ôD«hÕ$Ì=üøñAI?øà½÷>qâÄ7ß|£nGÅ¯gÎd@@wJÐ*ZE«fDnnnjjj\ÄðáÃå´Õ®];Év¿Lµvvv£Fbc¢Õ«¡´´´ûöÚéÅ£¼>Zäêàà0nÜ8­[·JÉ'|ÒÍÍM.CCC/]ºÄ>B«hõïÏ Õ:¦¨¨(##CN[111'Oí/')''§Ì;×ËËë¡ÊÌÌ,))ÉËËË¼1tÔVkM6ðÁcLäªùð¸!/ÀÞA«hõ_qní!ªÛ³gOllldd¤âN9%Ip)æ´iÓ$_ÎVÚ»@ÒÑÑÑl707­G;uêÐ¹sçGyÝVÑ*ÔDEË/>ºS¡ÞÞÞFEEÅÇÇïÚµË@×2°lF0C­W´þå_N>Í¾@«hj7Jp):ô÷÷wª 00P$*vQWt'`É%"`îâÙjÐ*Z¡"KMjee%R!fjjêÕt§±©­ZËC%|ÔC%1mÚ´¡ÕÁ§ÀL´ºoß¾ÁÜu×]k×®UùreyõD*--ýøãÙhî KHºzõêØÚÚjÂÐÚ"%ðåÞ/V·nÝêèè¸yóæ6DGGÛÙÙ-[¶Lò>pïÝ»÷úë¯go¢UÞ[µpÆ+§Ùñññ%%%uú·±²bYiµcÇÁ?üÐÁÁA?üðÃK/½TVV&KrÇwÈE¿mÛ¶ÅO2åöÛoÿöÛo¥Ìm·Ý¦z.×ë_®ã[¶lùßÿþWÌwÁ#G|ã7ØÝh­6H222ÂÃÃ]]]½½½£¢¢êë6,ZSiuÓ¦MOí.Y²ä«Òê'äÍÉÉ¿1B®;ÝÜÜRSS?ûì³&M:tè>äêÕ«çÏ/	ñëÆ[·núôiýîø?ÿüsJÔ5kdF¯¼òý ¢ÕLãì"//oÑ¢E¢RùO6íúÝ­è	L¥Õüü|Í§vå2ñ«ÒêùóçÅvGQZÁ½÷~ñÅÏ=÷¸SVoºé&)yêÔ))©ÞÀÿØQ¿;~ÍM`ýQ¢U???v4Zmð4Ö§%%%IIIâ0;;»#G&$$x´.1þ5Vë`F:ZïfÍÉPi511Q¢Ì	&¼ñÆÎÎÎßÿ½hõ;îýõæl9`ÀÑª~wü­ê­Þu×]ìh´ñD¥r.ð÷÷_½zµ¹]IpÌP«rê+,,Ü½·ÄS§N-¯xÈ*Z4iÒ3dðÀM6Ý¼y³hU®S+Õª~wüìÐ¡ÐVÑ*;ééé-òôôtww7oJx"Wßêþª÷A´æ¦UõVkkkqªêõ÷£>­þðÃnnnúôéß¿ß¾/_®ÑêßÿÝ¤IVõ»ãÿ hUª?êý÷ßG«hÌüüüØØXùKx:yòäM6iFÌäd,iùU5êPÄÏ^óÑjµ¯¨óîøKJJÎ?_é(@«¢¢"Yù¯nÜ¸1,,ÌÉÉiÔ¨Q			úkÇEV­¢ÕÚb×®]á¨¨(	û¢££t­«é2­ZE«¦$44´A/Eô)ËïçççêêaLZ´hÐ*Z­­OÂ»zX½'ãàà nöÿ7­Ã7ß|3|øðnÝº1bË-Ù°aÃÜ¹sõ;8q¢ê5Iô'VÑªY#kXË:kÖ,M%B®Á2hÐjµüñÇ6662»üqñâÅÎÎÎÖ/¶jÕªî¹G¿£`//¯íÛ·ë6IÂh­6"JJJbccçÕbSooo'''¹àÝ¿ÿÕ U@«)¶jÕêÌ3j0**jß¾*0aÂc=¦VUGÁ2øÞïÝ~ûí3gÎtwwôÑI&åää¨b111!ZëuhþÇ=<== Î«=³&%%]ø<[ËÖjzzºæ/³qãÆ+µ|ùrÍUòðáÃíííÇ·bÅõ.i×®Ý~(9"]ÉTZUDlØ°¡CÎ3ÇÊÊJ´º`ÁÉk>øàaaaªØ¯¿þºmÛ¶­[·Êãþûï×ï½V;¡¡¡«W¯®ã¯¾ U@«µ¤Õò7SÅO=õøòàÁ?þø#<¢Æ9rÙ²eÚZ¬¥d;;;Ñª|òÉï¾û®¬¬LSO>Ð AÅÅÅúÝ³Ñjã¥¨¨HbSWW×E5¡*¸	­U Agll¬Æ¯cÆtòäÉÏ=÷Ê|è¡äï¯­U¹¼Tc»víª­®_¿~Ò¤I=Z£ÕW_U¨X¿[`ö2Zmøùù]Mó"Ñèèh°°°×M?Z´Z-6l¦¦¦Sÿøã___&%³oß¾gÏ;ÆÇÇkkUxîÙ³GÝ?þ³Ï>[^ñDVÝ=böÔ|øða5#ýnÙËhµá!×¡¸ÉÝÝÝÅLWÓn­³VÅ;88´¨àá...¾xñâôéÓ¯»î:Éì±ÇÄ¸¢U	CUGÁrµ-A­£££KiiiGUÝqKùåË«b7ÜpCÓ¦MÛ¶mÛ¦CétÌ^n0Z5¼·µ0­úûû×¬»ú2«ªâ(<[´j<'NÐyÄSTAUå%î,--ÕùÇéäTz¥[à¤Õ#GøøøÈ5êÍÉÉüöíÛûúúê¿heyZõº¢. ä_?jÔ(¹!5 Ç¨*6åèèèM6^Fù£Z´ZC²téÒ²²²ùóçë:ujxx¸D«/¿üò°aÃC´jâNRTTÔ<<<&O,B5/~_«W¯öôôÌÈÈÈËËK¹òG´hµ&HXfoo¯³-[¶èxè¡FqáÂ¹sçN>½¼¢#ÿæÀD«éééË/pÖÉÉÉßß_¢ºýû÷7¬ðT_«aaaü-­Z5ÉÉÉnnn*Õ¼ys;ø¹¹¹¢N:ùùù)ÈQk¥µµµ¥î0uwÚ´i®®®îîîrm!¾Ó°ÈÉÉÙµkK@«VMÃúõë»té¢ÒÙÙÙM4ÑéÅãµ×^2dÈO<!ñèÂË+Ñïý7r^¶°hU4<<¼W¯^rU1räH	L333t`Z)²ïÇhÌW«gÏµ··WíÐÒÒÒ|µÇÚØØN$ýÁHºÒ®³,æÙê=æÎèàà ¿bËS©6Ü´hÕÄJ%1sæÌ9sæH¢¬¬lÙ²e¾páÄj_õdnÞ¼YlyZÍÍÍ]¾|ùäÉ]]]eeoºé¦Á«þMhÐ* Uììììãããååuüøñò[ VVV+W®´üvêÔÉÛÛ»[·nUÝ-lpZÕÜãõóó³µµ7oúÄwÃÕ»»»$%%Éº¸_¦ÚÁ   Q£Fñ·´hÕ=z´ª>$ÿØ±c&o(ZMOOV÷x=<<¦O¾víZÀ´ájURTTTPPyc-¦ùZE«9kU´!îSzzzJ`*Yll¬L+E8MwØ;ÃhÐjÃÐjZZÚ¼yóÄ4¢RooïÔÔÔ+ê;©Á!a·e·®@«Vë±cÇª¬CBBÏNÕf­ZE«5DÐ¤¤$ÕøÈÉÉ)00pÉ%³ï=www´h­¢U´ª«ÉL#HKKvpp¡Î5kÓ¦MsW­cIII^^f«D«h­¢U]âââ¬¬¬Ü«ÃÃÃ#,,,66ÖätÄÍõß855UmY5¹nÐ¬rµ4Y@«h­êRïï·Ôûðq´h­¾"V­¢UCß÷hÐªhµÞ»à&0Z´j9Z­wÐ*Z´V"###55µ¼¢¹oJúh­ZE«F1jÔ(+«ÿÁÁÁHBg&KhÐ*Z5Y5YAþhÐªåk5$$$**ªVg1yòd´V­Z¾VgÍåíí]Ûý+XöWtÐ* U´Z¾víZWW×:èù/222))ÿZ´jÉZ 2''§fÄ³U´hÕòµj$Ø7Ï»DÀ£3heeVÐ* U´Z=îîîiiiµ*ôôtþhÐ*Z­^«|î­ZmÔZM­­ U@«hÕ¨;´&©¯ U@«hÕdZ§p| U@«hÕp­Z­S­N6ÍÛÛ;Ð VVV£Fª¥åh¿¿JJ&'­Tåáá¡8::zÓ¦MjÉÑ*Z´ZwZ§JhRµ±ùùùvvvqqqyyyW_[pp°fi¥B¦cB´hµî´*!v¤XÇ,Y²Äqp||<2Z´Úxµ*Ñª	[êªÏ¦Z´ÚHµjâ½VÐ* U´VÐ* U´jvL6C­ZmZ7yG4Y@«V£Vsss]]]M®Un U@«Q«³fÍ2ý^D«hÐjcÓj~~¾ª999h­ZE«WKIIÉ=j£f, U@«N«µMÐ* U´jº½ÈM`´h­¢U´h­^1µýQq´V­6"­¥¦¦Ö^ý4Y@«VVwíÚÕ«W¯ZVi²V­6­®]»¶v÷"7Ð* ÕF¢Õ¨¨¨ZUÑ*Z´Ú´Z­ U@«hÕTÐd	­ZE«&&KhÐªåkµ¶©þÿ½ÈM`´hÕ²µºdÉ´V­¢UÄ©îîî			h­ZE«WK||¼§§gÝ¦ÉZ´ZÏZõóó«=­ÆÄÄ$%%ÕÍú¦§§×Ù¼­Z­#rrrîjÊ*,Y²D.ÜÝÝ%Á¡V­ÖV%Nõöön 6Ý¸qcXXXAAÁ¬Y³Ö®]+	c´hµ>µ*Bhp«íêêWgn­Z­F«½zõJKKk«·dÉ	LwíÚËQV­V333jo®¾Û#±éþýûE¥îîîÓ¦MKMM%<@«VÍQ«111µÝQÃÕ¼H*aôôéÓíìì"""J*à0@«VÍW«ÁÁÁqqqúù-òôôªX`5­ÖlÚüü|??¿%Kp³­ZmZ-((°µµÍËË«T«)))©©©2(¿)Ô`P´Zãi­Zm0ZMJJ¸°ÒQê¶L>c­¢U´jùZ«ªóµzõj	@«VÕª»»û=Ð* U@«W«Õ^½zdÑ* U@«Æj5<<Ü@çJÑÑÑ6mâ´hÕ(­zzzhmkÂ&KWß U@«f­Õ½+ð&0ß´VÍK.[VVV­ÆÄÄ8LV­¥iõÈ#>>>£G.,,ÔûçÞ÷ÝmÛ¶urr¼"­¾7V­¥iuÈ!K.x4$$dþüù:cÇsòäÉsçÎ×jAAØp&l²DwVÑjýSTTdoo¯¾-³eËí±þøã"NMæ|0ñßêk5!!ÁÏÏÏðÜMØd	­¢Õú'99ÙÍÍM¥³²²7o~áÂÍX	%E«Ãwvv4hÐöíÛ%ówÞùoF¡¯ÕiÓ¦-Z´ÈðÜyoÐ*£V322j6áúõë»té¢ÒÙÙÙM49wîfìD«üñ¥K~úé¡CVZI¥7t®V­YkUÔØ£Gsrr®hÂ³gÏÚÛÛW|vÔ××Wì3gD´ò+é;w¶lÙ²Ò&ÁUi533­Z©U«DoS®Y³¦  ÀÈiE¥9sæ9sÊ+^§Y¶lÙáÃ%Ý¿ÿØØØòæEU¼ÆZ5a%º´VMÆ#G^xáooo«ËHdùÀ|ûí·_9-¯x¼êìììãããååuüøqÉ)))V®^qØÎÎ®÷îþþþ÷î5­VMØdl­¢UÓóÛo¿-°yóæ¿·lÙbxªâââ£GVÕ#X3++ËÀäæp­ZE«¦¤´´tóæÍS§Nurr²ú7mÚ´ÑnßkrÐ* U°(­FDD´oß^ãQggçÙ³g8p`ÇöööóóÏ?[¶VéÐ*Z5ªÉRÓ¦MG¨Ý»ý÷Ýg¶ZåÃpVÁµ:zôèW_õôéÓú£RSS7lØP\lZ¥%@«`Z½ã;¨#Aj«V­´ûv0C­òÞ* U0#­nÛ¶mYÍ5[v7Þx£[·nVVViiihÐ* U£Ø½·øÌªjªÅhî ­¢U0gÎ=ÚØØX[[÷¼L>ôæoÖÍ*C%^°´VMÆ¸qãV_«T¿M¢¢¢Ð* U´zµ¤¤¤¬[·îÏ?ÿÜ¼yóºÊ¸xñ¢9kµf7Å£ªbm­[h­¢Õ«¢wïÞ¥%''kºÚ×¡ÒWnºV¥Z©#	Ð*X«·Ür££ã?ü0`ÀÇÊ@­Z´zeìÝ»·ªòÍY«5k²V­BíjµK./½ôRÝÜõ5VkÖdIçÙ*ZE«&¦k×®êajÓ¦MÇ÷í·ßÖeðJwVÁ¢´zâÄÅ÷èÑCÓXIâ×_|Ñ"[ U¨]­jØ¹sç¤I,»%0ZZ×jiiéî½÷Þ-Z(§^íµÿý·9kÃZsÔêóÏ?ïââ¢	RõêsæÌºY%,ZÒªêÂÖÖöøáêxxoÐ*XVïºë®èèè¿þú«^V	­ZKÐjãì­ZZÑjCï¸fMx¶hjE«½O`|­¢U÷V­Eiõ;î8p vÎ÷Ý×ªU«sçÎ¡U@«VbÛ¶mË*pttlÖ¬Ù²Ë¼ñÆÝºu³²²JKKC«V­ÅîÝ»ÅgVUS­ØêW«4Y´f¤UaÎ9=ö´±±±¶¶îy>ú4èÍ7ß¬Uªã&K¼`hjK«qãÆ6¬¾V÷V­%hî Ð*Z5½;ig«V¡V´ÚÐ»àÃpVÁ´j&ÐË U°4­îÞ½[,UZZºzõêaÃÍ9³nú(ç½U@«`aZ95mÚtÕªU+V¬Ð<X½óÎ;-I«»víJ¹LQQG U¨-­^ýõâÑeË8P¾îºë$qòäIÑjhhhàe¸uhjK«¥¥¥bµüü|ÕéÒöíÛöYIlÛ¶ÍµJ%@«`vZ-++³±±	¶nÝZDûðÃK:;;ÛµJ%@«`vZ¼¼¼4iÒ¦MQéøñã/^,	ºY¥Ú»	¸ÿ~@«P§Z]·n£££¨´U«V»wï^¸p¡XÖüû6¬ÕÔÔT©¤¤¤Ð*Ô©VóçÏ'&&?~;vìØ¾­R-iuäÈK,á´õ Õßÿýë¯¿þìßðÌY«,ùûûçççsÄZºÖê»ï¾Û¤I×'0M­9jµsçÎ"Qkkë¶mÛ:kaæÓË U0;­ÚØØ´oß>''§^V	­ZVÐ¥KúZ%´h,J«|òÕ1cÖ®]ôÕeÌü3æ6Y[¾|9 U¨7­6ÐÏWÚd©W¯^iii(V¡Þ´Ú¯_?ûÊhpM$x­rZúÔjýbB­ÆÅÅM6£Ð*Ô³V÷ïß?qâDÛo¿Û¶mË-kZ@«PÿZÝºu«¦;__ßèèhI<þøãf®U>hÌQ«¢RñèèÑ£ûõë'é/¾øÂÎÎ®U«VuÓO=½,ZËÑjiiióæÍ¯»îºK.M0A´*ªhwîÜiÎZå&0 U0ÇhµM6M6=pàÒêüÑ½wÑjVVZ´hõÊUV%lµ©@Ò]»v­U¢%0 U°(­þý÷ßÒîÂÍÍmïÞ½f®Uý&KbYq-G	 U¨O­*6oÞ,¢zíµ×<X«dÂ&KhÐ*Ô³VKJJÎ;§ÒYYYkÖ¬Ù°aCQQùkÀVÁ´ZZZúßÿþ÷Úk¯õÕWeðí·ßÖÜnß¾µV3C­ U¨7­¾õÖ[J¢,8|ø°¸MÒêW0`Z´hÕXúöí+ú2dÈöYIÛÚÚfdd|ùåÊ¬Ãæ©UzY´f¤ÕV­Z;ûí7Ißxã¾ç4VÁ;v³Vée	Ð*V[·n-îÌÍÍýã?Ô½ß·Þz«¼â«£££ùkÀVÁ´ªÞUâ'&N(	kkëãÇFFFªÀuÒÐ*XV.]ªÝÄØ±c%sèÐ¡jpÔ¨Qu³J&Ô*ï­ZzÓªðÌ3Ï´oß¾Y³fâÔ¿ÿþ[rÆ#N@öäÉf®UzY´æ¥U¡¬¬ìâÅÁ-[¶ìÜ¹S2¯¨K.Õ½Võ,qÐ*Ô³V¯#Gøøø8::=º°°°Ò2ë×¯÷òòªªç&,Z´ú2déÒ¥ÝÌ?_¿ÀÉ';vìheeuþüyÌÏÏÏþ7GE«V­Kjoo¯ÂÐ-[¶èÝÞzë­¯½öZÓ¦MVå¨µÒÃÚÚ­ZÆ®Õääd777ÎÊÊjÞ¼ù´¼ðÂ#GF«R,íßlÝº^­ZýßCÓ.]º¨tvvv&M´û;Ü¾»Ä²"Î_ýÕÆÆfß¾¶¢%@«VÿÇÙ³gE¥¥¥úúúj]ºtizõêeee%¿v2LwV­þ¢ÒÄÄDIÌ9sÎ9åÏS-[vøðaMÂÂBM¥ZÕ*ï­Z­Õäädggg//¯ãÇW|]$ºråJ­ê<vE«V­VBqqñÑ£GkÜ#	Yâ&0 UhðZ½Jh²hÐjýk÷V­ZE«V­¢U@«V-X«ô²hÐªÉ´ªi²heeÂQhÐêkUs¸¤¤$555¥@«V¯X«&Lxûí·9&­Z5V»wï>iÒ$	@«VM Õo¼QõBV­^­VÇÃ1hÐª	´Ú·oß3fpLZ´zÅZ¶µµÔäÐ U@«5ÔªH4??­Z´zµZHOO×Î¡%@«Vk¨Uø0 U@«&Ó*7­ZE«V­¢U@«V¨VmmmsssµsÒÓÓ´sh²hÐjõZ¡ÚÙÙéLIIÑÎ¡É U@«ÕkuãÆþþþÕjÀV­V¯ÕQ&Z´hÕZ<yrtt´N1ýî Ð* U@«ÕkuÀ©©©ÕNH%@«V«ÑjQQvß¿UA%@«V«Ñjzzº»»»1rÐ* Õj´ºvíÚQ£F¡U@«VM ÕyóæEDDèÓï­Z´ZV%T×/¦ÿM­Z­F«îîîû÷ï7F«4Y´hÕVlmmKJJÑ*7­Z5¤ÕÔÔT??¿JÑ U@«W¦UÑdhh¨¢U@«ViuÖ¬YK,1rB,Z´jH«þþþÆ&KV­Òªñ¦ä&0 U@«UjU¢OªÑ U@«W Õ#GVUl­Z½­.Z´(<<¼ªbÞÞÞIIIÁÁÁH&KV­V©ÕÐÐÐ¸¸¸ª988GSSSS*0æ¬h¯V%ÝµkWUÅ®»îº5kÖpZ´Z½VKJJªê¶P¡ÿl­Z­iii­(ÉhÐjõZ3PLBÕüü|@«V«×jµÝrÐ* Ucµ:räÈ¤¤$Q7nt¿Z´hÕ(­ºººæääè*))É¼¿¿?Z´hÕ(­:99.F%@«VÕjPPvfBBBAAvM­Z5J«ÖÖÖÓ§O×Îtww×Mi²hÐª±ZE«V­@«VVVÉÉÉEEEJ×]wÝáÃ5ùùùhÐ* Ucµ¤yÆßßÿã?ÖFFFÒd	Ð* UcµÊ®´h­Z´VÐ* U´hõµªßZ´ZC­ê¿·V­¢U@«VÑ* U@«£ÕÈÈH:Ö´hÕ4Z@«VÑ* U@«hÐ*ZE«h­jõÒ¥K5KwV­þGñññqtt=ztaa¡ÎØûï¿ßÝÝ½S§N3fÌ(--5^«¼`h£V²téÒ²²²ùóçëËÏÏ?uê³³³ hÐ* Õ*)**²··_IoÙ²%   ª^°`$>ùäGþÍ)SÐ* U@«åÉÉÉnnn*Õ¼yó.èûþûï[·nàÀI¿õÖ[CÿÄ»thÐjùúõë»té¢ÒÙÙÙM49wîN;vtìØñë¯¿®ªZZ´ú?Î=koo¯Ú"¥¥¥ùúúêØ½·³³óæÍTV­Zý?D¥9sæ9s$QVV¶lÙ²ÃwëÖmÁÿTpE-Ð*4F­&''K<êãããååuüøqÉ)))M®ò_~±Òâ©§B«V­VD¥G5ÜçèÐ* UÁ«V­¢U@«VÑ*ZÆ¦Uº´hÕdZ@«VÑ* U@«hÐ*ZE«h­Z­^«thÐª	´*6ÍÌÌ´³³ã@«V¯V«Ë/wwwÜÜ7 U@«W¥U´h­Z´VÐ*ZE«hÐ*Z´h­Z´VÐ* U´h­Z´VÐ*ZE«hÐ*Z´h­Z´VÐ* U´h­Z´V­¢U´VÐ* U´hÐ*Z´h­ U@«hÐ*Z´h­Z´VÐ* U´hÐ*Z´h­ U@«hÐ*Z´h­Z´VÐ* U´hÐ*Z´h­ U@«hÐ*Z´h­Z´VÐ* Uv- U@«hÐ* U´V­ U@«hÐ* U´hÐ*Z@«VÐ* U´hÐ*Z@«h­ U@«hÐ* U´hÐ*Z@«VÐ* U´hÐ*Z@«h­ U°T­^ºt©ÆcÑ* U@«ÿÇ#G|||G]XXxEcÑ* U@«ÿbÈ!K.-++		?~µcE®yÿæÄhÐ* Õò¢¢"ùô-[ª+G­UeØ6UÐÖ´iÓ¦ìPvh£I&ÁÁÁh­Ö'ÉÉÉnnn*Õ¼yó.üoV¬X!ZÝ±cÇiKgäÈwß·Å¯æ¶mÛdÊÅ¯éØ±co»í6_ÍÝ»wËýúë¯-~MCCCo½õV4Vëõë×wéÒE¥³³³åZïÜ¹sFÕ þ´¢[ßycÆ¹÷Þ-~5>,;ô_~±ø5³ð]wÝeñ«ùûï¿ËÝ¹s§Å¯é)SfÐjröìYûÒÒRI§¥¥ùúú?­¢U´VÑ* U]D9sæ9s$QVV¶lÙ29·V:­¢U´VÑ* Õ*INNvvvöñññòò:~ü¸äÈpåÊE«h­¢U´hÕÅÅÅG­ªÏÃcÑ*ZE«h­Z5ñöÁ<ú´Åï¼·ÞzËâWóäÉ²Csrr,~MW¬Xñúë¯[üjþùç²CåâØâ×tÕªU/¿ü2A«Ð¸µz5³nM-	ÃkZVVÆ¡kI+Ûxl´Ú¹ú>-`5ï¿ÿ~ww÷N:Í1C½d©kªX¿~½êoËR×ôÏ?ÿ¼ûî»Û¶mëääi©«#ùíÛ·÷õõÝ¾Þ¡?ýôSXXü·nÝZ³ÐªYp¥[äjÆÅÅåçç:uÊÙÙ9!!ÁwhyÅ£Ö;ZYY?Þ×488xÌ1²²çÎ3²¥®æÔ©SÃÃÃ%ùåzë´´´Ï?ÿÜÞÞ¾R­ZÌ¹,5èCØòVSÁ/X°ÀRwhyÅMÑ[o½õµ×^kÚ´iÖªá5ÍÌÌëüñþ±à¨ðÐC1âÂsçÎ>ºRÛµk§¯U9åkµ[Þjjøþûï[·nàÀKÝ¡Â/¼0räHI4t­^ÓM6Vîìì<hÐ w´ÚëääÔ©S'??¿KÕªÅÀòµj>új*vìØÑ±cÇ¯¿þÚw¨ØE.ùÓÒÒ~ýõWûö5Ü=×tÃ¢Õ?þøÒ¥KO?ýôÐ¡C-r5×^mÈ!O<ñÄ5×páBKÕªÅÀòµj>újWô!aÍæÍúáhxM.]Ú§^½zuä·á¯é3gäÌ+¿Þ¹sgË-èáÕ,,,Ë£ÌÌLIðÁ¶ÙTªU9åkµÜ7ôÕ,..îÖ­Ûþ© ¡·6¼C5§ch²dxMû÷ï+èèè.899õÕW)"ÓªEÀòµzõ7ôÕüå_´¿ßþÔSOYðÕÖjC:exM7lØ`gg×½wÿ½÷ZêjÊo§N¼½½åÒ0..®AïÐ·ß~»cÇ×Ü÷Ýz.Ë×jùU÷!l«iI°¦þù'++ËâWSò;ÖÎ¶çÀF«VÐ*Z´VÐ*Z´VÐ*@]q¡MéÒ¥ÌÌÌãÇ×Ù_dGýë¯¿ØqhÀìHNN¾¦+°°°ðþûï·³³S¸öÚkÃÃÃÿùçÓ.ØÚÚÚZêß±cGyÅ·ÛÚµk§æøÊ+¯h2frbþ×Ä­2;vì(Ö©Vï¸ã¥7[[[¥.!""ÂäZU5§¥¥Éà< é¶mÛÞyçßÿ½ö(c&ôgÆ®@«&fÕªUÊ:WªÕóçÏ7kÖLÅÅÅ¹¹¹¾¾¾2(6ùrîÞ½ûçVß|¹<ýôÓú£ºh­:Mo¾ùæhõôéÓÊÇsæÌ)((mÛ¶=óÌ3/¼ðBIIÉ¹sçzUß¯_?	gýýýùåÍ´÷Ýw½½PPÐT~aaá¬Y³ÜÝÝ[´h1tèÐnöîÝ[ªÚ·oÄ2JfÚ¾ÉÔeÌä^ËjÊä;w:uêðáÃ%ÿ7ÞPsß¹s§JaÚ U nûé§¶nÝZ³ÀbJeV	[²xñâßÿ]úûï¿Õ(kkk	ÌÚ´ióçtöì©n×%Ñ²eË'NÈT·ß~»ÊÕÕU%>ýôSí»¸Ý»wWUÉ´RÎÞj'ïÓ§fïºë®§zJÒ^^^jEÉ2xË-·p` UcX«/#:Ô%.ÔL£«O>ùD[«cÇ-**:tèPÓ¦MePÔûÑG©2yii©hLÃÃÃwìØ¡&Y·nÔ°`Á®]»>þøã:î4h¤_zé¥ò?75rríÀéééjÑ.]êÔ©¤ß÷]	´P+ZÕ´	7o^éä¡.]ºôîR¬I&gÏÕh511QSÏD|ðÁIØÛÛ¯ànÁÛn»måÊ°³³Óñ·Z5rrg«ª¶gyæ~PÏ;Ç!VêZ«_|ñEppðÐ¡C%U9?ýô*¹aÃVÔX),¡¡¡â0uã7PI&-[¶LMU(¿ªZ5rr­Jl*×Î9S-ÇZ¨-­^¸páÀe:¤=jãÆJWQQQÅÅÅ#¾öÚk*gß¾­N8ñÒ¥KêõÖùóç§¦¦*­J=¢ÃÊ2$''«I¾ùæÉýõ×­­­¥fc´jää...þè£ÔZhÌÆÆF~7oÞÌñVjK«pP½Q£n½¶nÝZ¥ûõëW®õlUèÑ£GÛ¶m%Ñ¢EÒÒR'''tss»÷Þ4i"þ#||Ôôïß_=ûí·Vsvww¿ï¾ûÔH ¬tîÜ¹Î:´EoW:áÉ''L *-µjÕjüøñgÏÕÖªHK½ÞÚ¾	pÕû÷ïïÛ·¯êA¢[·nW³³¦ò»ví:öì²TUª$Õüøå_ÖÖªeÌä¯¾úª*Ð«W/5SõTU;w.Z¨g$lýí·ßDiÚ­:t¨¨¨èèÑ£.]ÒP,Á«~YYY5^j'eÓ.ðáªE=|ø0­#ÚZ5Ûüì³ÏT´Ä^@«fJ~~þµsøþûïkþfdd°×Ð*ÔÒÒÒììì'Nèß ´V­ U´h­ U´h­ U@«h­Ô*'Od;Ôÿý÷ê²fv. U°L/^^ÁSO=%é­[·jõÕW×­[W_ËÖ«W¯éÓ§³ê+VXYYÕÆç`«ªÙT;·~Q@«ºtíÚÕÝÝüøñvqq3à)SÕØAnÑjµì¬­ÖVu6uý¢V*ÑêC=¤Ò/^3-ZdËV3¯ÉÅ`d7ß|ó>X¡6Tgr­ÐäZ­vS×Í´P½V²²²Þ½KØªï¼óÎ¨¨(ÄM7ÝÔ¼yó>úlÚ´Ie;v,((¨E'OîÞ½»:©åääÜsÏ=m*3fÌ'4>ÿüó³gÏvvvîÛ·ïæÍT^Õ÷«¯¾ØºY³f:u:räÊÌÊÊ>|¸­­­ß			OcÞ¼y?þ¸£££×®]»ÞxãÎ;_ýõ~ø¡+]þ9sæÈ·jÕJê«j	µ©t¥ò¹sçÞwß-[¶%¯t´òÏ>û¬ì>YøÕ«WWZOUûâ÷ßW§GcÇÕÈÏßß?&&Fyýõ×TúøñããÇo×®,¶¬©º¥QéBVU³V§Nª³ðRÕ´iÓ4e&MôÿüG*ýM­Vº0Õî´µ¨U!""BÎýõØ$ýì³Ï<(§Ý!CHNiiiÏ=o¸áõë×ÿðÃýúõS'PÉ´R×­['g7Iôïß_­*´±±¹í¶Û>ÿüóo¼qàÀUU^V¥r9³?òÈ#bµ3gÎ¨«Êúé§3f¨ËªOÕÜ¤IÏÆe´mÛÖ××÷Ë/¿1b«ª°ªåuÉµLûã?>ºÒ%Ô¡ªõµ¶¶8q¢ °°°ÒÐ1Ó5×rúôÓOÅ:õHN¥ûB¶äKÉo¿ýö»ï¾»k6NäCÍ"22R'oooÙP¯¾úê¡Ce5+ÝJj®váçÏ/?þøC¹YöQRRöT:Zû ©ê¨v_ U¨]­.^¼XÎ*¦Ñî~úi	$½mÛ6)ùÎ;ïèÜîÛ±c$dPå/_¾%"TJèpñâEI¿øâÐTUyUZú;vì(n«&3--MÝ»EÓ´¤÷îÝ[Õâ©G©Ò¹ºº;wNÒï¾û®¹páB¥X~í;.a¥è¯opp°5Ò7Ó< Ò2_|ñN=Uíq$V®¿q*ÕªZððpí¨t!ÔÂK`-*åW$S¶º3pXsTº0Æï´µ¥Õ)S¦ØÙÙémÙ²eruvvîÒ¥2A||¼¼~þùgèÇ¬/'qÂ:FGGk´¤_¹ÀríÞ½»Ô)jT£LÛó2Vµx:5K(#qJ¯Y³FiµÒ,¿Î¹^	u¨v«Z3iÊK4)ÁßÜ¹s+­G_(ñìÞ½ÛH­ªz$üÕ^JÒ@ÍÆ,¼l1OOÏââb2.¿éªÒjU[¬ÚV¡µúç¶nÝzüøñ:ç¬-[¶hB%K(<yÒÚÚú½÷ÞSçÌ£N [·nÄûï¿¯òW­Z%Û·o¯JKV^n°UÌENß-[¶ÐD*±±±ÚeªZ<c´Ziµª½*]BmYßª ªùå_¤¼£:ùUíUþóÏ?WùK.ÕÖê3Ï<£ò'L ´*He>hUg!ÔlÌÂùåÒ´iÓÜÜÜJµª½©u¢ÕJ·VÁôZ3fÌ±cÇöîÝûÑGuëÖM¢ãÇë³äµnÝ:9Mûûû7iÒDÝ5½é¦$ä³í¸qã$ÆI¦4H±Jµ~~~ªK¥ZªªòJµ*ÎSgvvv^^^§NTõäOo¾ùFf$3ýý÷ß«Z<c´ZUUi544T6]~~~UK¨1ë[Õè©GÊ&N(£:XÐ®ÇÀ¾¸îºë$Û¶mrå!ÊÑÈï[n8p ~òÉ'%SólµO>...bèS§NüÎ?_ÕBVU³1_ZZ*M&]Vé«½©µW¶Ò©j_Ì=;,,¿? U¨­ZUÐ¬Y³=ÊyGVÍØÞ½Ï1C³*Å$á®½öZOOÏò&$Ú0`Ö¬YÏ>û¬CÕrRÚTÍûöÝ·oNå­L¥kÖpæÌë¯¿^Ù¼yóaÃiÌÌÌ©DFµiÓ&11ÑÀâi×,Zí×¯V«ª°ÒåWÁÄ U-¡#×·ÒÐ1IM¨SOUûB®¢Df)Çv(/3jß¾½Ô$õ(­Wôäëë«êéØ±£Èµª¬ªf2dþÂ«;Ï2Irrr¥G¬ö¦ÖYYý©j_ÈvuäïhêÓ§O$!ÑÕÙ³guÆ4¨ÿþÚ9üñ¶¤¯¦r$X©´dVÕ%þâõï4JT$Áá%¼Òõ5°*P+++ÓoUTº/þùçÍû6ÚxQ5×GÂ>ýQúYUÍÚGEEE.üÔ©So¸áã7uµSí¾@«`.ÜÿýwÜqÇÿû_c$&PíX¼ºÁ";wmK¤ÍÐ*4F¾þúëç~âÄO=õÔ¶mÛX¼ºä­·ÞZ¿~½­Ô¾ûî¹¿ÿþ? U´h­ U´h­ U@«µÎÿ2Ó.°>~hIEND®B`
